# Detection of Leishmania infantum DNA in the non-parasitized lung of dogs with visceral leishmaniasis

**DOI:** 10.1186/s12917-018-1730-7

**Published:** 2018-12-17

**Authors:** Ricardo Goncalves, Soraia Oliveira Silva, Gregório Guilherme de Almeida, Carolina Carvalho de Souza, Wagner Luiz Tafuri, M. Norma Melo

**Affiliations:** 1 Departamento de Patologia Geral, Instituto de Ciências Biológicas, Universidade Federal de Minas Gerais, Av Antonio Carlos 6627, Belo Horizonte, MG CEP31270-901 Brazil; 2 Departamento de Parasitologia, Instituto de Ciências Biológicas, Universidade Federal de Minas Gerais, Av Antonio Carlos 6627, Belo Horizonte, MG CEP31270-901 Brazil

**Keywords:** Leishmania infantum, Leishmania (Leishmania) chagasi, PCR, Dog, Histopathology, Immunocytochemistry

## Abstract

**Background:**

Despite the very low or absent parasitism in the lungs, the interstitial pneumonitis is a common lesion found in humans and dogs with visceral leishmaniasis. The lung is a neglected organ in the study of dogs and humans with visceral leishmaniasis, but interstitial pneumonitis represents an important lesion characterized by thickening of the alveolar septum due to fibrosis and inflammatory exudate, and its pathogenesis is still uncertain. In this study, the polymerase chain reaction (PCR) was used to detect Leishmania infantum in paraffin-embedded lung biopsies from naturally infected dogs from an endemic area in Minas Gerais State, Brazil; PCR was compared to histological and immunohistochemical techniques for detecting Leishmania.

**Results:**

Eighteen dogs in which leishmaniasis had been diagnosed by serological tests - indirect immunofluorescence assay (IFAT), enzyme-linked immunosorbent assay (ELISA) and complement fixation tests (CFT) - were classified as asymptomatic, oligosymptomatic or symptomatic. Nine of the 18 dogs studied had a positive PCR (50%) but parasites were not detected by histopathological and immunocytochemistry methods.

**Conclusions:**

These data indicate that PCR on DNA extracted from paraffin-embedded tissue is a valuable method for detecting Leishmania infantum parasites in lungs of naturally infected dogs, despite the apparent absence of parasites from standard HE (hematoxylin and eosin) stained slides and of labeled parasites from immunocytochemical preparations.

## Background

American visceral leishmaniasis is a severe systemic disease caused by *Leishmania* (Leishmania) *chagasi* (= *L. infantum*) and transmitted by the bite of the sandfly *Lutzomyia longipalpis*. It is an important public health issue in many countries from South and Central America, particularly Brazil, where over 90% of reported human cases in America occur [1]. Dogs are known as the most important reservoir of this disease. The prevalence of canine infection in American continent varies greatly; in Brazil it is higher than elsewhere and generally precedes human cases. The clinical and pathological aspects of canine visceral leishmaniasis are highly variable and lesions appear to be a consequence of damage induced by both parasite and host immune response. Clinical signs of the illness in dogs are numerous and vary considerably. Most frequently reported, clinical signs are generalized lymphadenopathy, loss of weight, onicogriphosis, alopecia, and exfoliative dermatitis with scaling, keratoconjunctivitis, hyperkeratosis, apathy and ulcers, paw edema, vomiting and epistaxis [2].

In a mammalian host, the amastigote form is located in the parasitophorous vacuoles of mononuclear-phagocytic cells, mainly in macrophages. The presence of amastigotes in lungs of dogs was first reported by Donatien [3]. He described the presence of parasites associated to a cellular exudate comprising macrophages, lymphocytes and plasma cells in the alveolar septum. Subsequently, some authors described interstitial pneumonitis in humans with the presence of numerous amastigotes within macrophages in the affected alveolar septa [4–6]. However, other authors found no significant presence of parasites in lungs from naturally and experimentally infected dogs [7–10]. Moreover, Duarte et al. [8] throughout an immunoenzymatic study (Peroxidase anti-peroxidase – PAP) of lungs from naturally infected dogs, using specific antibodies, demonstrated particulate material and rare amastigotes in the inter-alveolar septa, despite the inflammatory process defined as interstitial pneumonitis.

Previous works has demonstrated that paraffin-embedded tissues are suitable for DNA extraction and for some downstream molecular methods [11–16]. Thus, the aim of this study was to detect *Leishmania infantum* by PCR in paraffin-embedded lung tissue from naturally infected Brazilian dogs and to compare the results with those from histological and immunohistochemical studies.

## Methods

### Animals

Twenty-two mongrel dogs, with undefined age, provided by the Zoonosis Control Center kennel of Belo Horizonte, state of Minas Gerais were used in this study and others [17, 18]. Prior to euthanasia, all dogs were examined to clinical signs compatible with canine visceral leishmaniasis. These dogs were divided in two groups. Four mongrel dogs, three females and a male, with negative serological tests for *Leishmania* - indirect immunofluorescence assay (IFAT), enzyme-linked immunosorbent assay (ELISA) complement fixation test (CFT) – composed the control group. Eighteen dogs, nine females and nine males, formed the group of naturally infected dogs. These dogs were positive to serological tests: indirect immunofluorescence (IFAT), enzyme-linked immunosorbent assay and complement fixation test (CFT). All these dogs had the parasites isolated from bone marrow and identified by isoenzyme electrophoresis as *L*. *infantum* (data not shown). Furthermore, these dogs were divided in three sub-groups and classified in symptomatic, asymptomatic and oligosymptomatic in agreement with the classification previously described [9]. Smears from organs as liver, spleen and bone marrow were also positive for *Leishmania* in all dogs. The Institutional Ethics Committee of Federal University of Minas Gerais, Brazil, approved this study.

### Histopathology and Imunocytochemistry

Dogs were anesthetized with 2.5% Thiopental sodium (1 ml/kg) and euthanized using a lethal dose (0.3 ml/Kg) of T-61® (Intervet). Fragments of lungs were collected randomly from the pulmonary lobules, during necropsy, processed and embedded in paraffin. Histological lung sections were stained by Hematoxylin-Eosin (HE) for the demonstration of amastigotes in dog’s tissues. Also, immunohistochemistry (IHC) was carried out on paraffin-embedded tissue samples (0,5 × 0,5 cm) stained by biotin-streptoavidin peroxidase immunostainning technique, in agreement with Tafuri et al. [19]. Fragments from spleen and liver with high parasitism, were used as positive control.

### DNA extraction

DNA was extracted from paraffin-embedded tissues blocks previously used for histological and immunohistochemical diagnosis. Sterile razor blades were used for extracting tissues from each block and to remove the excess paraffin before weight. 5–10 mg from each sample were placed directly in 1.5 mL microtubes and crushed with a glass pestle. The crushed fragment was deparaffinized with three xylene washes and washed twice in ethanol. Special precautions such as using new disposable razor blade, gloves and bench-top paper for cutting each block were used to avoid possible cross-contamination with *Leishmania* DNA. After deparaffinization, DNA was extracted using Genomic DNA Isolation KIT Puregene® following the manufacturer instructions, with some modifications. Briefly, the material was submitted to lyses for 15 min at 65 °C, using cell lyses solution. Samples were then homogenized using a pestle and submitted to additional incubation at 65 °C for 30 min. Following incubation, the material was digested with 1.5 μL of proteinase K (20 mg/mL) overnight at 55 °C. Subsequently, the sample was incubated at 37 °C for 1 h in the presence of 1.5 μL of RNase (7 mg/mL). The mixture was subjected to protein precipitation, DNA precipitation and hydration following supply recommendations. Finally, the DNA was stored at 4 °C for at least 24 h before used.

### Polymerase chain reaction - (PCR)

Serial 1:10 dilutions (1:10, 1:100 and 1:500) of each DNA sample extracted as above were used as templates for PCR amplification of a 120 base pairs (bp) fragment of *Leishmania* kinetoplast DNA (KDNA) minicircles, using oligonucleotide primers 13A (5’-GTGGGGGAGGGGCTTCT-3′) and 13B (5’-ATTTTACACCAACCCCCAGTT-3′) described by Rodgers et al. [20]. PCR were performed in a 25 μL reaction volume using 1 μL of each diluted DNA, 0,8 Units of Taq Polymerase (CENBIOT-RS-Brazil), 200 μM of each dNTP (PROMEGA, Madsion, WI, USA), 1.5 mM MgCl_2,_ 50 mM KCl, 10 mM Tris-HCl and 0.5 pmol of each primer. Samples were overlain with light mineral oil and initial denatured at 96 °C for 6 min. Cycles consisted of annealing at 50 °C for 45 s, extension at 72 °C for 1 min and denaturation at 94 °C for 30 s, in a total of 26 cycles. Positive controls were made using spleen or liver DNA from dogs positive for *Leishmania* in parasitological tests, also embedded in paraffin blocks and 1 ng of DNA from promastigotes of *L. infantum* strain MHOM/BR/1976/BH46. Tubes without template DNA and lung extracted DNA from control dogs (negative in sorology and parasitological tests), were used as negative controls. Amplification products (10 μL) were analyzed by electrophoresis in 5% polyacrylamide gel and stained with silver salts [16]. To ensure that negative results corresponded to true negative sample rather than to a problem with DNA degradation or inhibition of the PCR, sample DNA was also tested using human β-globin primers. All PCR tests were confirmed by a repeated amplification.

## Results

**Fig. 1:**
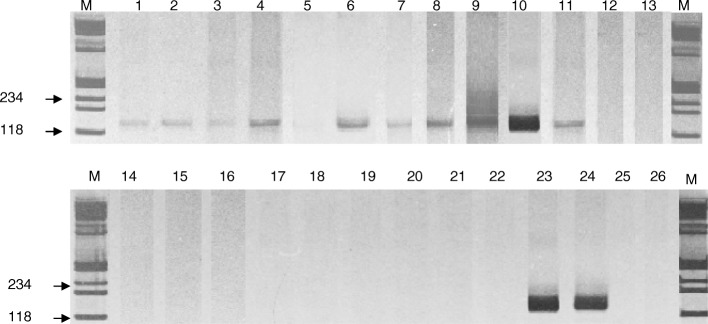
t represents 3 - Gel of polyacrylamide 5% stained by the silver, showing amplified products (PCR). M = Marker of molecular weight (fX174). - Numbers 2, 3, 4, 5, 9, 21, 22, 23, and 25: dogs naturally infected with positive reaction. - Numbers 6, 7, 8, 24, 26, 27, 28, 29, and 30: dogs naturally infected with negative reaction. - Numbers 14, 15, 33, 34: dogs controls with negative serology for leishmaniasis. - Numbers 10, 11, 12, 13, 16, 31, 32, 35: they are positive controls

**Fig. 2:**
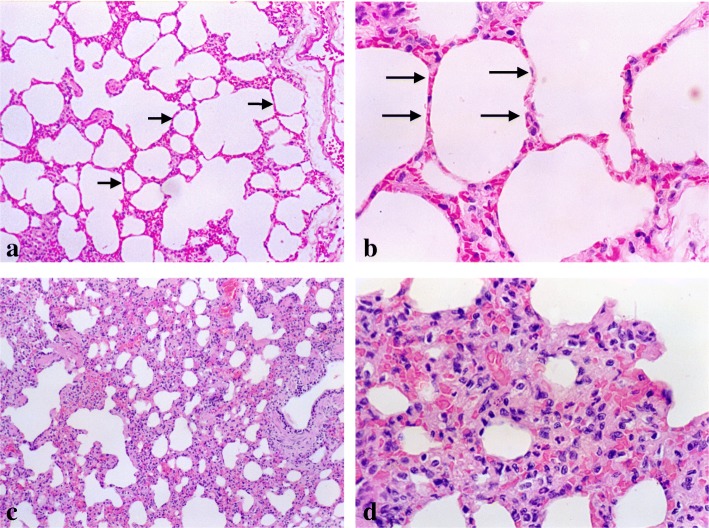
**a, b** Paraffined lung section of control dog. Note the normal alveolar septa. HE. 110x and 440x; **c, d** Paraffined lung section from a naturally infected dog. Note the thickened inter-alveolar septa in the naturally infected dog with an intense and diffuse chronic interstitial pneumonitis. An intense septal thickening associated with a severe and diffuse chronic interstitial pneumonitis is observed. HE 110x and 440x

Serial dilutions of DNA extracted from paraffin embedded lung tissue were able to amplify the expected products in nine (50%) of the 18 naturally infected dogs. Figure 1 shows the visualization of the amplified products of all the samples with their respective controls. DNA from lung of control dogs and intrinsic internal control show no amplifications. Although the molecular findings showed the presence of DNA, no parasites were detected in the same lung tissue analyzed by the histopathology (HE) and immunohistochemistry (IHC) techniques. However, a chronic interstitial pneumonitis with a remarkable thickening of the inter-alveolar septa was observed, in all infected dogs, independently of clinical group (Fig. 2c and d). The cellular exudate was mainly composed by macrophages, lymphocytes and plasm cells (Fig. 2d).

**Fig. 3:**
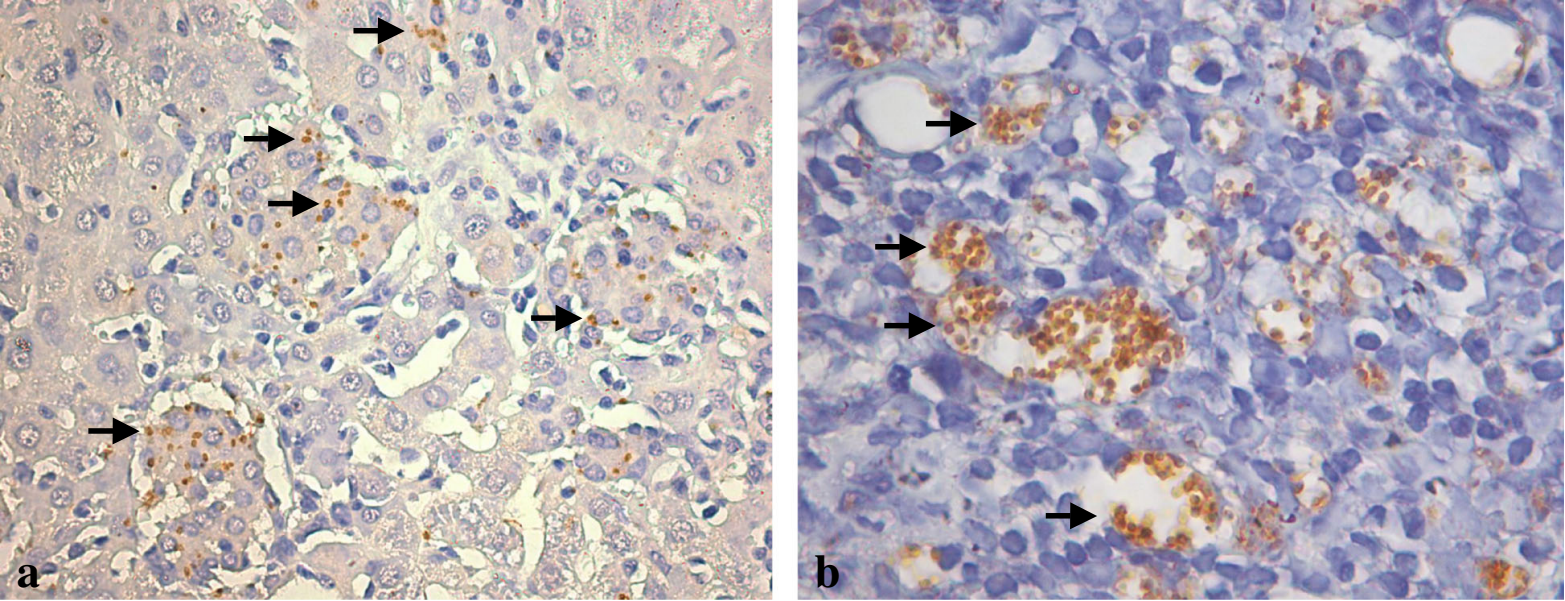
Liver and spleen sections from naturally infected dog. Immunohistochemistry analysis of liver (**a**) and spleen (**b**) from a naturally infected dog, showing the intense parasitism. Stained amastigotes are shown inside infected macrophages (Arrows). Immunohistochemistry – 440x

The positive control of PCR reactions was carried out considering livers and spleens samples from one naturally infected dog. These organs had an intense parasitism as we can observe in the histological and immunohistochemical study (Fig. 3). DNA samples isolated from liver and spleen of infected dogs, also embedded in paraffin, were serial diluted 10-fold and were subjected to PCR as described above and the sensitivities of the PCR were correlated with the presumed number of parasites in the infected organs.

## Discussion

PCR of DNA extracted from paraffin embedded lung tissue was able to amplify the expected products in 50% of the naturally infected dogs examined, despite no evidence of parasites in the same fragments of lung tissue analyzed by the histopathology and immunohistochemistry techniques. This finding suggests that there were amastigotes or *Leishmania* DNA in the lungs. The amount of DNA required to amplification was previously investigated by serial dilution. It had been observed that the major amount of DNA (non-diluted DNA) produced no results (data not shown). It occurred, probably, because there were inhibitory PCR factors in the tissues.

In our study we used a large amount of material for DNA extraction (5 to 10 mg), compared with those used by other authors (3 to 4 fragments of 5 μm). Our choice is justified by the difficulty in ascertaining how much paraffin contributed to the measured weight and because the fixation and embedding procedures themselves could cause relative degradation of DNA. Also, we have not found parasites through hematoxylin-eosin (HE) and immunohistochemical (IHC) methods. Therefore, as the expectation of finding positive samples was very low, we tried to increase the amount of material, improving the probability of detecting Leishmania DNA. In addition, a positive control was carried out using fragments from paraffin embedded livers and spleens, from one naturally infected dog. These organs had an intense parasitism as we can observe in the histological and immunohistochemical study (Fig. 3). Otherwise, the detection frequency of parasite DNA is different in distinct organs from naturally infected dogs [13].

In recent studies, we observed that is possible to use different amounts of material for the extraction (5 mg up to 150 mg), without losing quality of extracted DNA (data not shown). It improves the probability of finding Leishmania DNA in materials with low parasite load or apparent absence of amastigotes. We consider that the inconsistency between molecular results and HE or IHC methods reflects the excellent sensitivity of the PCR method. Indeed, in all of our samples, analysis by optical microscopy and immunohistochemistry were not able to detect parasites in the lungs. Probably two slides of 4 μm cannot be considered significant when it is an organ with low parasitism. Thus, PCR has shown more effectiveness, perhaps by the higher amount of material which increases the probability of Leishmania DNA detection. Moreover, the results could be reproduced several times in different dilutions.

The fact that 50% of the samples were negative, doesn’t mean however that there is no parasite DNA in these lungs, since many factors may have influenced this result. Some authors have been concluded that PCR method might fail due to wrong tissue-fixation protocols [18, 19].

On the other side, the absence of parasites in the lungs observed under HE and IHC analysis in all samples from infected dogs, is in accordance with literature information about canine visceral leishmaniasis [7, 8, 17, 21]. Duarte et al. [8], for example, showed peroxidase positive particles (PAP) in fragments from lungs tissue characterized as immunolabelled particles or amastigotes in the interstitial of the thickened septa lungs. Otherwise, in human visceral leishmaniasis some authors described the presence of few amastigotes in lungs from humans, but never in an exacerbated way [4, 5].

In spite of the negative results, under the HE and IHC analysis, no correlation was found between the presence of specific clinical signs typical of canine visceral leishmaniasis and positivity of PCR. However, a chronic interstitial pneumonitis with a remarkable thickening of the inter-alveolar septa was observed, in all infected dogs, independently of which clinical group they belonged to. It is in accordance to Gonçalves et al. [18] results, which have shown that chronic interstitial pneumonitis coexists with lesions of other organs. In addition, cellular exudation and a peculiar fibrosis are the mechanisms responsible for the remarkable focal and/or diffuse thickening of inter-alveolar septa.

## Conclusion

In agreement with the results obtained in this work we could conclude that the method for DNA extraction from paraffined fragments is satisfactory. We obtained a suitable yield and material quality for the PCR. In addition, the PCR was shown to be more effective than the histological and immunohistochemical methods, to detect parasites in tissue. These results have been confirmed by Xavier et al. [13], who demonstrated that PCR is more effective method than HE and IHC to detected amastigotes in canine paraffin-embedded ear skin. Thus, these data indicate that PCR, carried out on DNA extraction from formalin-fixed and paraffin-embedded tissue specimens, is a viable method for detection of *Leishmania* also in lungs, especially in chronic cases where there is low parasite load in the organ. However, we cannot discard that repeated negative results of PCR were due to phenol and/or chloroform, which can inhibit the *Taq* polymerase enzyme, or the present of other inhibitory components [22] generating false negative results.

## Abbreviations

CFT: Complement fixation tests
DNA: Deoxyribonucleic acid
dNTP: Deoxynucleotide
ELISA: Enzyme-linked immunosorbent assay
HE: Hematoxylin and eosin
IFAT: Immunofluorescence assay
IHC: Immunohistochemistry
PAP: Peroxidase Antiperoxidase
PCR: Polymerase chain reaction
